# Application of Advanced Emulsion Technology in the Food Industry: A Review and Critical Evaluation

**DOI:** 10.3390/foods10040812

**Published:** 2021-04-09

**Authors:** Chen Tan, David Julian McClements

**Affiliations:** 1 China-Canada Joint Laboratory of Food Nutrition and Health (Beijing), Beijing Technology & Business University (BTBU), Beijing 100048, China; tanchen@btbu.edu.cn; 2 Department of Food Science, University of Massachusetts, Amherst, MA 01003, USA; 3 Department of Food Science & Bioengineering, Zhejiang Gongshang University, 18 Xuezheng Street, Hangzhou 310018, China

**Keywords:** nanoemulsions, high internal phase emulsions (HIPEs), Pickering emulsions, multilayer emulsions, solid lipid nanoparticles (SLNs), multiple emulsions

## Abstract

The food industry is one of the major users of emulsion technology, as many food products exist in an emulsified form, including many dressings, sauces, spreads, dips, creams, and beverages. Recently, there has been an interest in improving the healthiness, sustainability, and safety of foods in an attempt to address some of the negative effects associated with the modern food supply, such as rising chronic diseases, environmental damage, and food safety concerns. Advanced emulsion technologies can be used to address many of these concerns. In this review article, recent studies on the development and utilization of these advanced technologies are critically assessed, including nanoemulsions, high internal phase emulsions (HIPEs), Pickering emulsions, multilayer emulsions, solid lipid nanoparticles (SLNs), multiple emulsions, and emulgels. A brief description of each type of emulsion is given, then their formation and properties are described, and finally their potential applications in the food industry are presented. Special emphasis is given to the utilization of these advanced technologies for the delivery of bioactive compounds.

## 1. Introduction

Emulsions are colloidal dispersions that consist of at least two immiscible fluids (normally water and oil), with one of them being dispersed in the other in the form of small droplets [1]. The principles of emulsion science and technology are commonly employed in the food industry to create a wide variety of emulsified food products, such as beverages, milks, creams, dips, sauces, deserts, dressings, mayonnaise, margarine, and butter. The nature of emulsions confers these foods with distinct functional attributes, such as desirable appearances, textures, mouthfeels, and flavor profiles. Moreover, emulsions are a widely used vehicle for the encapsulation and delivery of bioactive agents, such as vitamins and nutraceuticals. Conventional emulsions are composed of numerous emulsifier-coated fluid droplets dispersed within another immiscible fluid medium [2]. The dispersed droplets typically have diameters between about 200 nm and 100 μm. There is considerable scope for varying the properties of conventional emulsions by choosing different oil or emulsifier types, by incorporating different additives (such as thickening or gelling agents), by varying their droplet size distribution, or by manipulating their oil-to-water ratio. As a result, different physicochemical, sensorial, and nutritional attributes can be obtained. However, conventional emulsions do have some limitations for certain applications within the food industry. For instance, they are susceptible to breakdown through physical instability mechanisms, such as gravitational separation and droplet aggregation. Moreover, they have a limited ability to control the release profile of encapsulated ingredients. In addition, high fat contents are often needed to form oil-in-water emulsions with viscous or semi-solid textures, which is a drawback for creating reduced-calorie versions of some products. Recently, there has been growing interest in improving the healthiness, sustainability, and safety of foods in order to address some of the negative effects associated with the modern food supply, such as rising chronic diseases, environmental damage, and food safety concerns. In this context, there has been a trend toward the exploration of more advanced emulsion systems that have novel or improved functional attributes, such as reduced calories [3], controlled digestion behavior [4], and increased bioavailability of bioactives [5]. These advanced emulsions include a broad range of soft complex materials, including nanoemulsions, high internal phase emulsions (HIPEs), Pickering emulsions, multilayer emulsions, solid lipid nanoparticles, and multiple emulsions.

**Figure 1:**
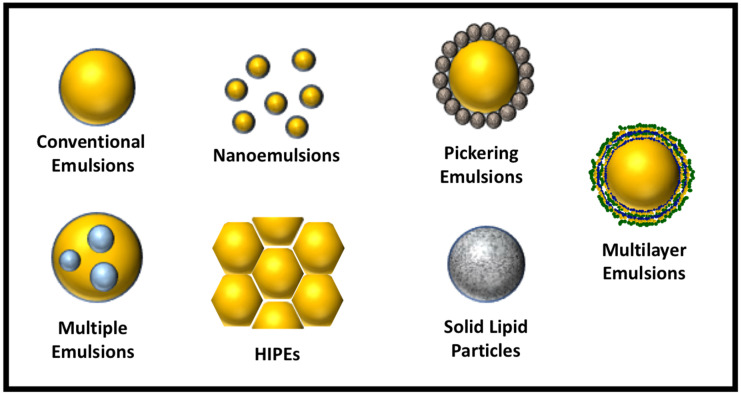
Examples of different kinds of advanced emulsion systems that can be designed using food-grade ingredients. HIPEs, high internal phase emulsions.

In this review article, we focus on the development and application of these advanced emulsion technologies. In particular, we focus on emulsions where the continuous phase is aqueous, since these are the most widely used in the food industry. The structures of these advanced emulsions are illustrated schematically in Figure 1. The key technical information from various literature reports is collected and tabulated, including the physicochemical principles underlying their formation, the choice of ingredients, the preparation conditions used, and their intended applications in foods. Special emphasis is given to the utilization of these advanced emulsions for the encapsulation, protection, and delivery of bioactive compounds. Finally, we discuss the challenges associated with the application of each of these advanced emulsions.

## 2. Nanoemulsions

Nanoemulsions are similar to conventional emulsions except that they have droplet diameters that are smaller, ranging from around 20 to 200 nm [6]. The small size of the droplets in nanoemulsions means that they have several potential advantages over conventional emulsions for specific applications, including better stability to gravitational separation and droplet aggregation, greater optical clarity, and higher bioavailability of encapsulated bioactive compounds [7,8]. The size of the droplets in nanoemulsions can be controlled by altering their composition and fabrication methods. Typically, the droplet size decreases as the emulsifier concentration increases, the oil-to-water viscosity ratio tends toward unity, and the homogenization intensity and duration increases. The droplets in oil-in-water nanoemulsions can be considered to consist of a hydrophobic core containing oil and an amphiphilic shell containing the emulsifier. The shell-to-core ratio of nanoemulsions is larger than that of conventional emulsions, which affects their properties and functional attributes, such as their creaming stability, optical properties, and rheology [9]. The methods used to fabricate nanoemulsions are generally categorized as either high- or low-energy approaches. High-energy methods involve the use of mechanical devices, including microfluidizers, sonicators, and high pressure valve homogenizers [8]. These kinds of devices are already commonly used in the food industry, are relatively easy to scale-up, and have a high throughput, which means they are suitable for large-scale commercial production purposes. Low-energy methods include spontaneous emulsification, phase inversion temperature, and phase inversion composition methods [10]. These methods utilize changes in the chemical potential of the components when the composition or temperature is changed in a specific manner. Although low energy methods have few equipment and operating costs, they require relatively high levels of synthetic surfactants and are often highly sensitive to changes in environmental conditions, which limits their application [11,12].

Nanoemulsions have been commonly explored for their ability to encapsulate, protect, and deliver hydrophobic bioactive compounds in food and beverage products. For example, whole-fat milk fortified with vitamin D nanoemulsions [13] or curcumin nanoemulsions [13] were shown to maintain good physical and chemical stability throughout storage. The encapsulation of resveratrol in nanoemulsions has been shown to significantly improve its resistance to chemical degradation when exposed to UV light [14]. Nanoemulsions have also been utilized to incorporate active ingredients into edible coatings designed to extend the shelf life of foods. For example, fortification using edible films with either ginger oil or curcumin/cinnamon oil nanoemulsions has been shown to extend the shelf life of chicken breast fillets [15,16]. Similarly, edible films loaded with citrus oil nanoemulsions have been shown to inhibit the growth of microorganisms on fish (silvery pomfret) during refrigerated storage [17].

Another promising application of nanoemulsions is to enhance the bioaccessibility and bioavailability of hydrophobic bioactive compounds [18]. In vitro studies using simulated gastrointestinal models have shown that nanoemulsions can increase the bioaccessibility of β-carotene [19,20], lycopene [21], resveratrol [22], curcumin [23,24,25,26], and 5-demethylnobiletin [27]. Information about the preparation procedures and droplet sizes of nanoemulsions used for different applications is summarized in Table 1. The observed bioaccessibility enhancement of these bioactives is linked to the small dimensions of the oil droplets in nanoemulsions. As a result, they have a relatively high specific surface area, which leads to rapid digestion of the lipid droplets by lipases within the gastrointestinal tract. On the other hand, the large exposed surface area of the droplets in nanoemulsions may have adverse effects in some situations. For instance, bioactive substances that are prone to chemical degradation when exposed to the aqueous phase may degrade more rapidly in nanoemulsions than in emulsions. Taking curcumin as an example, it is highly unstable to chemical degradation when exposed to neutral or alkaline conditions. Recent experiments showed that curcumin encapsulated within nanoemulsions (180 nm) was degraded more rapidly than curcumin encapsulated in medium (520 nm) or large (13.6 μm) emulsions, which was attributed to their higher surface area [28].

In vivo studies have also been carried out to evaluate the effect of nanoemulsion encapsulation on the oral bioavailability of certain bioactive compounds. For example, the impact of droplet size on vitamin bioavailability and bioactivity was assessed by orally administering mice with vitamin D_3_ nanoemulsions, vitamin D_3_ coarse emulsions, or blank nanoemulsions (no vitamin D_3_), and then measuring the serum 25(OH)D_3_ ((25-hydroxyvitamin D_3_)) levels using a radioactive immunoassay [29]. Compared to the blank nanoemulsions, the coarse emulsions increased serum 25(OH)D_3_ levels by 36%, whereas the nanoemulsions increased them by 73% (*p <* 0.01). This study therefore highlighted the ability of nanoemulsions to increase the oral bioavailability of oil-soluble vitamins. Animal feeding studies have also shown an improved bioavailability for other hydrophobic bioactive compounds delivered in nanoemulsions, including capsaicin [13], vitamin E [30], and coenzyme Q_10_ [31].

Despite their ability to increase the bioavailability of hydrophobic bioactive compounds, the application of nanoemulsions does encounter some practical challenges. Due to the large surface of nanoemulsions, a relatively high concentration of emulsifiers is often needed, which may increase production costs or generate off-flavors. The relatively thin interfacial layers and small droplet dimensions in nanoemulsions limits their capacity to control the release of encapsulated bioactive compounds. In some cases, the ability of nanoemulsions to greatly increase the bioavailability of hydrophobic bioactive substances may be a challenge. For instance, some studies have shown that the consumption of high levels of vitamin E or β-carotene can promote cancer in certain populations, such as smokers. Consequently, more research is required on the behavior and toxicological effects of nanoemulsions within the human gastrointestinal tract.

## 3. High Internal Phase Emulsions

High internal phase emulsions (HIPEs) are concentrated versions of conventional emulsions, typically having an internal phase volume fraction (*ϕ*) that exceeds 74% [38]. At these high concentrations, the oil droplets are tightly packed together and may adopt non-spherical shapes. As a result of the tight packing of the oil droplets, HIPEs typically have semi-solid textures, which is useful for some applications. HIPEs are commonly stabilized using high levels of molecular emulsifiers [39] or colloidal particles [40,41]. Due to increasing consumer concerns about having synthetic ingredients within their foods, there has been a movement towards the identification and utilization of natural emulsifiers to form and stabilize HIPEs. Colloidal particle-stabilized HIPEs, also referred to as Pickering HIPEs, typically exhibit a higher resistance to calescence and Ostwald ripening than molecular emulsifier-stabilized HIPEs, which can be attributed to the irreversible adsorption of the colloidal particles at the oil-water interface [38]. Recently, a variety of food-grade colloidal particles have been explored for the preparation of Pickering HIPEs, including globular protein nanoparticles [42,43,44,45], protein microgels [46,47,48], cellulose particles [49], gelatin [50], chitosan microgels [51,52], starch granules [53], chitin nanofibrils [54], and protein-polysaccharide complexes [55,56]. The stabilizing effects of these particles are affected by their wettability, size, charge, and flexibility, as well as by the ionic strength, pH, and temperature of the surrounding solution. It should be noted that many food-grade colloidal particles have challenges to their commercial application. For example, polysaccharide-based particles often lack surface activity, whereas protein-based ones often suffer from structural dissociation at the interface [49,57,58]. Consequently, all these factors should be carefully considered when developing successful food-grade Pickering HIPEs. Particularly, the wettability of most food-grade colloidal particles is poor, and the three-phase contact angle (θ) cannot meet the requirements of forming stable Pickering HIPEs. For this reason, natural colloidal particles are often treated by surface modification or complexation with other biopolymers. For example, the θ of pure zein particles is around 68°, suggesting that they are preferentially wetted by the aqueous phase. After modification with propylene glycol alginate and rhamnolipid, θ becomes 84° so that they are suitable as effective stabilizers for Pickering HIPEs [59]. In another study, zein particles were coated with chitosan to obtain an intermediate surface wettability, thereby resulting in the formation of complex particles that can form Pickering HIPEs with good long-term stability [60]. A similar modification was performed for gliadin through complexation with chitosan [61]. More information on the utilization of food-grade colloidal particles for Pickering HIPEs has been summarized in a number of recent review articles [62,63,64].

Surfactant-stabilized HIPEs can be prepared using low-energy methods that involve the slow addition of the dispersed phase (oil) into the continuous phase (surfactant and water) under constant agitation, so as to avoid phase inversion. However, this is a time-consuming process and requires the use of synthetic surfactants, which severely restricts the large-scale production of HIPEs. Pickering HIPEs can be formed by directly mixing the oil and water phases together by hand-shaking, vortex-mixing, or stirring. More recently, protein-stabilized HIPEs have been formed using a one-pot homogenization method that involves shearing mixtures of oil and aqueous protein solution together for short times [42,65,66]. It is worth mentioning that the low-energy emulsification methods often generate large droplet sizes in the range of tens to hundreds of micrometers, which may increase the possibility of extensive coalescence, syneresis, and creaming.

**Figure 2:**
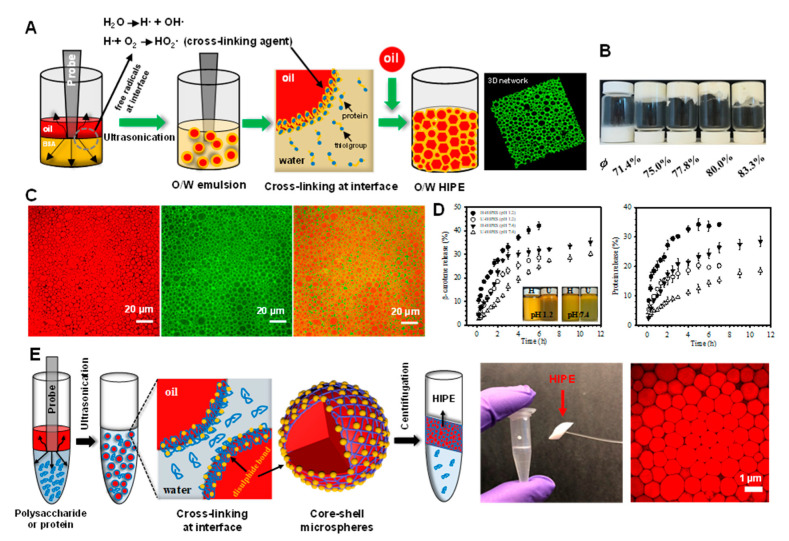
(**A**) Schematic illustration of the preparation of HIPEs by ultrasonication. (**B**) Photographs of emulsions prepared by ultrasonication at different volume fractions of oil (*ϕ*). (**C**) Confocal laser scanning microscopy (CLSM) images of HIPEs, showing the oil phase stained red (left), the protein-rich aqueous phase stained green (middle), and the merged images (right). (**D**) Release profiles of β-carotene and bovine serum albumin (BSA) from HIPEs during incubation under simulated physiological solutions at pH 1.2 and pH 7.4. The inset shows the visual appearance of the HIPEs taken after 6 and 12 h incubation at pH 1.2 and 7.4, respectively. HIPEs prepared by ultrasonication and homogenization are labeled as U-HIPEs and H-HIPEs. Reprinted with permission from [67]. Copyright (2018), American Chemistry Society. (**E**) Schematic illustrating the preparation of polysaccharide- and protein-based HIPEs through successive ultrasonication and centrifugation, right corner is the CLSM image of HIPEs stabilized by chitosan and pectin. Reprinted with permission from [70]. Copyright (2018), Royal Society of Chemistry.

HIPEs containing relatively small oil droplets (around 2 μm) can be prepared using more intense homogenization methods, such as sonication [67]. A cross-linked layer of bovine serum albumin (BSA) is formed at the oil-water interface during acoustic cavitation (Figure 2A). At sufficiently high oil concentrations, the resulting HIPEs have gel-like properties that are able to support their own weight, even when the vials are inverted (Figure 2B). Confocal laser scanning microscopy (CLSM) analysis shows that the HIPEs contain closely packed oil droplets surrounded by proteins (Figure 2C). The cross-linking of the BSA at the oil droplet surfaces prevented the proteins from detaching from the oil-water interface even when the HIPEs were placed in buffer solutions with pH values ranging from 1 to 11. These HIPEs were shown to have the capacity to control the delivery of model hydrophobic bioactive compounds (β-carotene) when exposed to simulated gastrointestinal conditions (Figure 2D). These results suggest that sonication may be a useful method of producing stable HIPEs. As shown in Figure 2E, after centrifugation at high speed, a stable HIPE layer with a high internal phase volume fraction (up to 89.5%) is formed [68]. The authors also showed that non-cross-linked proteins could be deposited back onto the oil droplet surfaces by adjusting the pH, thereby generating a thicker interfacial coating that increased the repulsion between the droplets [69,70,71].

Owing to their relatively high fat content and tunable viscoelasticity, HIPEs have attracted great interest for functional food applications. One important application of HIPEs that has been explored recently is as an alternative to partially hydrogenated oils (PHOs). Hydrogenation is usually carried out to convert edible liquid oils into solid fats; however, it leads to the production of trans-fatty acids that are detrimental to human health. For this reason, HIPEs have been investigated as an alternative strategy for creating solid-like textures in fatty foods. As an example, Pickering HIPEs stabilized by gliadin particles or gliadin-chitosan complexes have been shown to have the potential to replace PHOs in processed foods [72,73]. HIPEs have also been developed for application in semi-solid condiments, such as mayonnaise and salad dressing [74,75]. HIPEs stabilized by cod proteins (φ = 85%) have been utilized as food-grade 3D printing materials due to their high gel strength, hardness, adhesiveness, and chewiness [76]. HIPEs have also been used as delivery systems for improving the stability and bioaccessibility of hydrophobic bioactive compounds due to their relatively high oil content. For instance, encapsulation of β-carotene within ovalbumin-stabilized HIPEs was shown to reduce its chemical degradation during heating at 95 °C for 5 h [66]. The bioaccessibility of β-carotene has been shown to be increased 5-fold when encapsulated in gelatin-stabilized HIPEs [77]. Similarly, β-carotene encapsulated within whey protein-stabilized HIPEs had a considerably higher bioaccessibility (36%) than that dispersed in bulk oil (13%) [78]. The encapsulation of various other hydrophobic bioactive substances in HIPEs has also been shown to appreciably enhance their bioaccessibility, including curcumin [55,79], lutein [80], polymethoxyflavones [81,82], and resveratrol [83]. The increase in nutraceutical bioaccessibility after encapsulated in HIPEs has been attributed to a number of mechanisms: (i) the gel network in HIPEs reduces the aggregation of the oil droplets during digestion, thereby increasing the access of lipase to the lipid droplets and increasing their digestion; (ii) the bioactive compounds trapped inside the oil droplets are protected from degradation within the gastrointestinal tract; and (iii) the high levels of lipids present in HIPEs lead to the formation of a large number of mixed micelles, capable of solubilizing the carotenoids [84].

There are a number of challenges to the widespread application of HIPEs in foods. It is difficult to prepare HIPEs containing small oil droplets using current preparation procedures. It is important to identify food-grade emulsifiers that provide high resistance to droplet coalescence because the oil droplets are tightly packed together for prolonged periods. The extremely high fat content of oil-in-water HIPEs is unsuitable for the preparation of reduced calorie foods. Finally, the gastrointestinal fate of HIPEs and encapsulated bioactive compounds should be investigated using more realistic animal and human feeding studies.

## 4. Multilayer Emulsions

Multilayer emulsions consist of oil droplets coated by multiple layers of charged polyelectrolytes. The assembly of multilayer emulsions is typically based on the layer-by-layer (LBL) electrostatic deposition method. Initially, an oil-in-water emulsion is formed by homogenizing oil, water, and a charged emulsifier together. This leads to the formation of an emulsion containing charged emulsifier-coated oil droplets. This emulsion is then mixed with a solution containing oppositely charged biopolymers, which adsorb to the oil droplet surfaces and form an additional coating [85,86]. This procedure can be repeated several times by sequentially depositing oppositely charged biopolymers onto the droplet surfaces, which leads to the formation of multilayer-coated oil droplets. The LBL assembly process can be monitored by measuring the increase in particle size and the change in zeta-potential after each successive layer is deposited [87,88,89]. A washing step is often required between each deposition step to remove excess biopolymers from the aqueous phase; otherwise, they would interact with the next oppositely charged biopolymer. However, this washing step can sometimes be avoided if the biopolymer concentration is carefully controlled to ensure that the oil droplets are fully coated, without promoting bridging flocculation.

The successful formation of multilayer emulsions depends on the combination of biopolymers used, as well as the solution conditions employed, such as pH and ionic strength [64,90]. A variety of proteins and polysaccharides have been utilized to assemble multilayer emulsions. Often, proteins are used to form the first layer because they have better surface activity than polysaccharides. Polysaccharides are then used to generate the subsequent layers because they can provide strong steric and electrostatic repulsion. A recent study showed that the multilayer emulsions formed with ovalbumin as the first layer and gum arabic as the second layer had much smaller droplet sizes and better stability than those formed with the opposite arrangement [64]. Other protein-polysaccharide combinations that have been applied to prepare multilayer emulsions include gelatin-chitosan [91], whey protein-alginate [92,93], lactoferrin-alginate [59,94,95], and soy β-conglycinin-pectin [96]. Multilayer emulsions can also be stabilized using two or more polysaccharides, one that acts as an emulsifier and the others that form additional layers. For instance, amphiphilic octenyl succinic anhydride (OSA) starch has been used to create anionic oil droplets, which were then coated with cationic chitosan to form multilayer emulsions [97]. Alternatively, small molecule ionic surfactants can be used to form the initial layer and then biopolymers can be deposited onto the surfactant-coated oil droplets. For example, saponin has been used to form anionic surfactant-coated oil droplets and then cationic chitosan and anionic pectin have been sequentially added to form multilayers [98]. In other studies, sodium dodecyl sulphate (SDS) has been used to form anionic surfactant-coated oil droplets and then chitosan-alginate or chitosan-pectin multilayer emulsions have been formed around them using the LBL approach [23,99].

Assembling multilayer coatings around oil droplets may have various benefits, including increasing the electrostatic and steric repulsion between the droplets, forming a dense biopolymer network around the oil droplets that modulates diffusion processes, and incorporating functional biopolymers at the droplet surface (such as antioxidants). These features can improve the stability of multilayer emulsions to environmental stresses, as well as being useful for modulating the digestion of the lipid droplets and the protection and delivery of bioactive substances. Some recent examples of the application of multilayer emulsions for the delivery of bioactive compounds are shown in Table 2. As an example, it has been shown that astaxanthin degradation during storage is 3- to 4-fold slower in chitosan-pectin multilayer emulsions than in conventional emulsions [98]. Similarly, chitosan-alginate multilayer emulsions have been shown to preserve the antioxidant capacity of curcumin during digestion better than conventional emulsions [23]. Moreover, it is possible to modulate the delivery of encapsulated substances by controlling the number, type, and sequence of polyelectrolytes in the multilayer emulsions, as well as the deposition conditions used [15]. For example, the bioaccessibility of β-carotene was 30.2% for lactoferrin-coated oil droplets, 35.3% for lactoferrin-alginate-coated ones, and 70.1% for lactoferrin-alginate-ε-poly-L-lysine-coated ones [59]. Conjugation of proteins with polyphenols can provide improved functionality in multilayer emulsions. For instance, lactoferrin-chlorogenic acid/(-)-epigallocatechin-3-gallate (EGCG) conjugates have been shown to improve the stability of multilayer emulsions and the β-carotene encapsulated within them [100,101]. It was also reported that the nature of the outer layer has a major impact on the stability of multilayer emulsions. As an example, gelatin-chitosan multilayer emulsions were found to have better creaming stability than gelatin-alginate ones [91]. In another study, multilayer emulsions with an outer κ-carrageenan layer had better pH stability than ones with a pectin outer layer [102].

One of the major challenges in creating stable multilayer emulsions is to optimize the composition of the system [103]. If the biopolymer concentration is too low to saturate the droplet surfaces, then bridging flocculation occurs. Conversely, if it is too high, then depletion flocculation may occur. Typically, this means that stable multilayer emulsions can only usually be formed at relatively low oil droplet concentrations (<5%), which means that they are suitable for application in dilute food emulsions like beverages, but unsuitable for application in concentrated food emulsions like dressings or sauces [99]. In addition to the biopolymer concentration, other factors also need to be optimized when forming stable multilayer emulsions, including biopolymer type, droplet concentration, pH and ionic strength.

## 5. Pickering Emulsions

Pickering emulsions are also referred to as particle-stabilized emulsions. Unlike conventional emulsions, which are usually stabilized by molecular emulsifiers (like surfactants, phospholipids, proteins, or polysaccharides), Pickering emulsions are stabilized by colloidal particles, which may be organic or inorganic. Pickering emulsions are typically much more resistant to Ostwald ripening and coalescence than conventional emulsions because of the extremely strong attachment of the colloidal particles to the droplet surfaces. Colloidal particles that can act as good stabilizers for Pickering emulsions must typically have certain attributes: (i) they should be partially wetted by both the oil and water phases; (ii) their surface potential should not be too high, otherwise they will not adsorb strongly due to electrostatic repulsion effects; and (iii) they should be considerably smaller than the target oil droplet size [106,107]. Numerous kinds of food-grade colloidal particles meet these criteria and can therefore be used for this purpose. Recently, there has been an emphasis on the utilization of organic colloidal particles, rather than inorganic ones, to form Pickering emulsions because they are more label-friendly. Table 3 summarizes some of the protein-, polysaccharide-, and protein/polysaccharide-based colloidal particles that have already been shown to be suitable for forming and stabilizing Pickering emulsions. These organic particles are assembled from ingredients that are generally regarded as safe (GRAS) by the Food and Drug Administration (FDA), and which are are sustainable, abundant, and economical. The functional attributes of these particles can often be tailored for specific applications by carrying out specific physical or chemical modifications. The ability of Pickering particles to stabilize mechanisms can be attributed to various effects: (i) the formation of a strongly attached and thick interfacial layer that can generate steric and/or electrostatic repulsion; (ii) the formation of a three-dimensional viscoelastic network of aggregated particles in the continuous phase that inhibits oil droplet movement; and (iii) a depletion effect that promotes oil droplet aggregation, thereby inhibiting their movement [108].

Protein-based Pickering particles include alcohol-soluble plant proteins such as zein [109], kafirin [110], and gliadin [46], and water-soluble plant or animal proteins, such as soy protein [111,112], quinoa protein [113], gelatin [114,115], ovotransferrin [116], whey protein [117,118], and β-lactoglobulin (β-Lg) [119]. These proteins can be converted into nanoparticles, microgels, or fibrils that are suitable as Pickering emulsifiers using various methods, including antisolvent, thermal, and pH precipitation methods. The complexation of proteins with phenolic compounds can provide additional functionality to Pickering emulsions. For example, the formation of gliadin-proanthocyanidin complexes was shown to promote the adsorption of the protein particles to the oil droplet surfaces, as well as retarding lipid oxidation during storage and in a simulated gut [13]. Similarly, β-Lg-EGCG complexes have been shown to have much stronger antioxidant activity than β-Lg alone, thereby providing greater protection for encapsulated lutein [120]. Similar results have also been reported for zein-tannic acid [121], soy protein-anthocyanin [122], and whey protein-EGCG [123] complexes.

Polysaccharide-based Pickering particles mainly include those based on starch, cellulose, and chitin [124,125]. Native starch granules cannot efficiently adsorb at the oil-water interface during emulsification due to their relatively high hydrophilicity. For this reason, starch is often modified by attaching hydrophobic octyl groups to the polysaccharide chain to produce OSA-starch [126,127]. Another major disadvantage of using starch granules for this purpose is their relatively large size (1–20 μm), which means that the resulting Pickering emulsions tend to be extremely coarse, with oil droplet diameters between around 10 and 100 μm. To overcome this problem, hydrolysis [128], milling [129], nanoprecipitation [130], sonication [131], and high pressure treatments [132] can be employed to generate smaller starch particles that can be used to form Pickering emulsions. Cellulose is another commonly used polysaccharide to prepare Pickering emulsions. In particular, cellulose nanocrystals and nanofibrils have received considerable interest due to their amphiphilic nature, high aspect ratio, large elastic modulus, and ease of modification [114,133]. In addition, chitin nanocrystals [134,135,136], chitin nanofibers [137], and chitosan-tripolyphosphate (TPP) nanoparticles [138] have also been shown to act as Pickering particles.

Colloidal particles formed from protein-polysaccharide complexes are often better Pickering stabilizers than those from proteins or polysaccharides alone [139]. This is because polysaccharides inhibit the precipitation of proteins around their isoelectric points and provide good steric repulsion between oil droplets, whereas proteins provide good surface activity and antioxidant activity. Protein-polysaccharide complexes can be formed via physical or chemical interactions between the two biopolymers. Protein-polysaccharide complexes can be formed through electrostatic interactions. For instance, a variety of polysaccharides have been used to coat the surfaces of zein nanoparticles through electrostatic deposition and therefore modify their wettability and surface charge, including gum arabic [59,140], chitosan [60], corn fiber gum [141], and pectin [142]. The resulting zein-polysaccharide complexes were then successfully used to prepare Pickering emulsions. Covalent protein-polysaccharide conjugates can be formed through various methods, including enzymatic, photocatalytic, chemical, and Maillard reaction crosslinking methods [139]. In particular, Maillard products are commonly explored for their potential application as Pickering particles, e.g., whey protein-dextran conjugates [111,143].

Pickering emulsions have been investigated for their potential to encapsulate and deliver various kinds of bioactive compounds (Table 3). A major advantage of using Pickering emulsions for this purpose is that the thick particle coating prevents droplet coalescence, retards lipid oxidation, and modulates digestion [144]. In a recent study, Pickering emulsions stabilized by kafirin nanoparticles were found to exhibit less lipid oxidation and higher curcumin stability under UV radiation than those stabilized by Tween 80 [145]. This enhanced stability was linked to the ability of the kafirin particles to form a physical barrier around the oil droplets, as well as acting as interfacial antioxidants. A similar phenomenon was observed for curcumin-loaded Pickering emulsions stabilized by zein-chitosan complex particles [146]. Encapsulation of curcumin in starch particle-stabilized Pickering emulsions has been shown to improve its chemical stability and increase its bioaccessibility under simulated gastrointestinal conditions [147]. Another study demonstrated that the extent of lipolysis in an ovotransferrin-lysozyme particle-stabilized Pickering emulsion was increased by around 39% compared to the extent of lipolysis for bulk oil, and the bioaccessibility of the curcumin was increased from around 16% to 38% [148].

As with the other kinds of advanced emulsions, there are a number of limitations that need to be addressed before the widespread utilization of Pickering emulsions by the food industry. First, Pickering emulsions need to be produced on a large-scale using cost-effective and reliable processing operations. Second, more information is required about the stability and properties of Pickering emulsions when present within real food products. Third, more details are needed about the release mechanisms of bioactive compounds from Pickering emulsions within the human gastrointestinal tract. Lastly, the efficacy and safety of Pickering emulsions should be evaluated using in vivo animal model studies.

## 6. Solid Lipid Nanoparticles

Solid lipid nanoparticles (SLNs) are colloidal dispersions containing particles with a solidified lipid core, coated by a layer of emulsifier molecules [156]. Structurally, they are therefore similar to conventional emulsions except that the droplets are fully crystalline in SLNs rather than liquid. The solidified lipid matrix confers SLNs with some potential advantages, such as better protection of encapsulated bioactive compounds, prolonged release, and alterations in gastrointestinal digestibility [157,158]. Typically, SLNs are prepared by homogenizing an oil phase and a water phase containing a hydrophilic emulsifier at a temperature above the lipids’ melting point. The resulting nanoemulsion is then cooled below the crystallization temperature of the lipid phase to solidify it. One drawback of this method is that the high temperatures involved can promote chemical degradation of heat-sensitive bioactive compounds. To reduce the loss of bioactives, a cold homogenization method can be used, which involves dissolving the bioactive in a melted oil phase, cooling it to crystallize it, milling it to produce smaller particles, and then adding a surfactant and homogenizing at a cold temperature to produce SLNs [159]. Other methods that have been used to prepare SLNs include sonication, solvent evaporation, microemulsion, double emulsion, supercritical fluid, and spray drying techniques [160].

Edible lipids used to construct SLNs should be crystalline at the intended temperature of utilization but should be capable of being melted so that the bioactive can be incorporated. A number of edible lipids are suitable for preparing SLNs, including glyceryl palmitostearate, glyceryl monostearate, glyceryl behenate, palmitic acid, tripalmitin, steric acid, tristearin, and waxes (Table 4). The lipid composition directly affects the characteristics and performance of SLNs as delivery systems. For example, glyceryl palmitostearate has a relatively high melting point (52 °C–55 °C), so it can be used to tune the release behavior of SLNs in the oral cavity [161]. Vegetable fat has a melting point (above room temperature, but not too high) that allows it to be used to encapsulate heat-sensitive compounds such as probiotics [162] and vitamin D_3_ [163]. The properties and performance of SLNs prepared by glyceryl behenate, tripalmitin, and stearic acid have been compared [164]. Increasing the length of the fatty acid chains was shown to increase the particle size of SLNs and their polydispersity, while also increasing their encapsulation efficiency for clarithromycin. Lipid composition also affects the in vitro digestibility of SLNs. SLNs formulated with medium-chain triglyceride oil and glyceryl stearate can be fully digested, whereas those formulated with medium-chain triglyceride oil and hydrogenated palm oil exhibit slower lipolysis kinetics and higher β-carotene bioaccessibility [165]. The reason is that the presence of monounsaturated long-chain fatty acids after digestion of hydrogenated palm oil-containing SLNs enhances the solubilization capacity of the mixed micelles for β-carotene. On the other hand, the use of lipid mixtures allows better fitting of bioactive compounds into the more imperfect crystalline structure [166]. For example, the combined use of glycerol monostearate and steric acid provides SLNs with a smaller particle size and better polydispersity [167]. When applied to encapsulating EGCG, SLNs have 8.1 times higher cytotoxicity against MDA-MB 231 human breast cancer cells than that of pure EGCG. Compritol 888 ATO is another commonly used lipid mixture for forming SLNs, which consists of different esters of behenic acid and glycerol. It has a relatively high melting point (~70 °C) and has been shown to increase encapsulation efficiency and curcumin permeability in in vitro studies [168].

Emulsifiers play key roles in the formation, stabilization, and performance of SLNs. In conventional emulsions, emulsifiers predominantly affect the particle size and stability of the colloidal dispersions by adsorbing to the oil-water interface, reducing the interfacial tension, and providing high repulsive forces. However, in SLNs, emulsifiers can also modify the crystallization kinetics and the polymorphic form of the crystalline lipids, which is important as these factors impact bioactive retention and particle stability to aggregation [169]. It is worth noting that the emulsifiers used should have the capacity to form a resistant layer around SLNs because the oil surface would change dramatically during the formation of SLNs via solidification. For this reason, a mixture of emulsifiers is often used, such as Span 85/egg lecithin [170], lecithin/Tween 80 [171], and Tween 80/Span 80 [33,172]. Recently, more attention has been paid to the use of biopolymeric emulsifiers as the food industry tries to move away from using synthetic surfactants. For example, SLNs have been formed using bovine serum albumin-dextran Maillard conjugates as emulsifiers [173]. The same researchers have also prepared SLNs stabilized by sodium caseinate/pectin-complexes for the oral delivery of curcumin [174,175,176]. Polyethylene glycol (PEG)-based emulsifiers have also been widely used in SLNs. These PEGylated emulsifiers can hinder lipolysis initiated by inhibiting the adsorption of bile acids in the gastrointestinal tract, and therefore controlling the digestion of SLNs [177]. Studies using a rat model confirmed that PEGylated SLNs significantly increased the bioavailability of curcumin [178]. Other biopolymeric emulsifies that have been used to prepare SLNs are summarized in Table 4, including sodium caseinate-lactose Maillard conjugates [179], gelatin-gum arabic complexes [162], whey protein [180,181], and β-lactoglobulin [182]. It is interesting to note that although chitosan has very weak emulsifying capacity on its own, it can be deposited onto the surfaces of SLNs through electrostatic interaction, which leads to better mucoadhesive properties and a higher oral bioavailability of encapsulated curcumin [183,184] and resveratrol [185].

The major limitation of SLNs is that they must be prepared at elevated temperatures to avoid crystallization of the lipid phase during homogenization, which causes losses of heat-sensitive bioactive compounds. Some SLNs also have a tendency to undergo polymorphic transitions during storage, which can expel encapsulated substances and lead to alterations in particle morphology that promote their aggregation. Additionally, the transport mechanisms of various SLNs across the gastrointestinal epithelial cell monolayer remain unclear [157].

## 7. Multiple Emulsions

Multiple emulsions, also referred to as double emulsions, consist of an inner water phase (W_1_) that is dispersed in the form of small droplets within larger oil droplets (O), which are themselves dispersed within an outer water phase (W_2_). Compared to conventional emulsions at the same dispersed phase volume fraction (either O or W_1_/O), W_1_/O/W_2_ emulsions have a lower fat content but a similar perceived appearance, texture, and mouthfeel, so they can be used as fat replacers. This type of emulsion can also be used to encapsulate hydrophilic bioactive compounds inside the internal water phase and release them in a controlled or triggered fashion [1]. The standard procedure used to prepare this type of multiple emulsion involves two steps: (i) a water phase and an oil phase containing an oil-soluble emulsifier are homogenized together to form a W_1_/O emulsion; (ii) this emulsion and a water phase containing a water-soluble emulsifier are then homogenized together to form a W_1_/O/W_2_ emulsion [188]. Note, to avoid the unwanted breakdown of the W_1_/O droplets, the second homogenization step should be milder than the first one.

In common with conventional emulsions, multiple emulsions may break down due to droplet flocculation, coalescence, Ostwald ripening, creaming, or sedimentation. However, they are also susceptible to other kinds of instability issues. For instance, the internal water droplets can shrink or expand, as well as being expelled from the oil droplets. Moreover, they have two distinct O/W interfaces, requiring different kinds of emulsifiers [189]. An oil-soluble surfactant such as polyglycerol polyricinoleate (PGPR) is typically used as an emulsifier to form the internal W_1_/O interface. PGPR provides stabilization by reducing the interfacial tension and generating steric repulsion between the water droplets [190,191]. The non-adsorbed PGPR molecules in the oil phase have also been found to play a key role in the stability of multiple emulsions, which can be attributed to their ability to increase the oil phase viscosity [192]. However, in many food products the use of PGPR is strictly regulated by international regulatory authorities. As an alternative, Span 80, Tween 20, lecithin, and their combinations have been investigated for their ability to stabilize the inner W_1_/O interface [193,194,195]. Water-soluble emulsifiers, alone or in combination, are typically used for the stabilization of the external O/W_2_ interface, such as Tween 20 [196], Tween 80 [59], quillaja saponin [197], OSA starch [198], gum arabic [199], bacterial cellulose [200], sodium caseinate [201], and whey protein [202]. Recently, a variety of colloidal particles have also been utilized to stabilize the O/W_2_ interfaces in multiple emulsions via a Pickering mechanism, including kafirin nanoparticles [203], whey protein-gum arabic complexes [193], whey protein-pectin complexes [195,204], whey protein-inulin complexes [205], and chitosan-gum arabic complexes [194]. The rigid layer of adsorbed particles formed around the W_1_/O droplets can inhibit both the exchange of emulsifiers between interfaces and film drainage between the droplets in multiple emulsions. Polysaccharide thickeners can also be utilized to stabilize multiple emulsions by reducing the mobility of the W_1_/O droplets in the external aqueous phase [206]. A recent study compared the stabilizing effects of three types of thickeners (sodium alginate, chitosan, and xanthan gum) and found that xanthan gum conferred the highest stability to multiple emulsions [59].

As mentioned earlier, there are additional instability mechanisms in multiple emulsions linked to the diffusion of water molecules between the internal (W_1_) and external (W_2_) aqueous phases, as well as the expulsion of entire water droplets from inside the oil droplets. Osmotic pressure gradients between the W_1_ and W_2_ phases are the major cause of water diffusion. Osmotic active solutes, such as salts and sugars, can be added to the aqueous phase to balance the osmotic pressure between them, and thereby inhibit the net movement of water in the system. This effect depends on the type and concentration of solutes used [207]. For instance, it has been reported that multiple emulsions containing NaCl had a higher resistance to breakdown due to water diffusion processes than those containing CaCl_2_, MgSO_4_, or glucose [59]. It is worth mentioning that crystallization of the oil phase may be used to increase the resistance of multiple emulsions to osmotic stress effects. A recent study generated osmotic stress by creating a sucrose concentration gradient between the internal and external water phases in multiple emulsions containing either liquid (soybean oil) or semi-solid (hydrogenated soybean soil) fat [208]. The W_1_/O droplets in the emulsions containing liquid oil swelled/shrank when the external sucrose concentration was below/above the internal sucrose concentration. Conversely, no size change was observed for the W_1_/O droplets containing solidified fat under the same conditions. Another approach to increase the stability of multiple emulsions is to gel the internal water droplets. For instance, it has been shown that gelling the internal aqueous phase using thermally denatured whey protein reduces the degree of osmotic swelling of the multiple emulsion droplets after dilution in a hypotonic solution, whereas this effect does not work upon hypertonic dilution [209]. In the case of porcine gelatin, its gelation effectively improved the stability of multiple emulsions to coalescence even at high salt concentrations (2 mol/L MgCl_2_) [210].

Multiple emulsions offer an effective strategy to protect hydrophilic bioactive compounds within the inner aqueous phase, such as anthocyanin [199,211], water-soluble vitamins [192], folic acid [204], gallic acid [59], and peptides [198,201]. They also have the capacity to simultaneously encapsulate hydrophilic and hydrophobic bioactive components. For example, gallic acid and quercetin have been encapsulated in the internal and external water phase, with the aim of improving the oxidative stability of the oil phase (a blend of olive, linseed, and fish oils) [212]. More examples associated with the delivery of bioactive compounds using multiple emulsions are listed in Table 5. Generally, multiple emulsions coated by protein nanoparticles or protein-polysaccharide complexes provide additional beneficial effects, such as improved long-term stability, more prolonged release of bioactive compounds, higher resistance to digestive enzymes, and better oxidative stability [213]. The external water phase of multiple emulsions can also be gelled to modulate their textures, stabilities, and functional attributes. For instance, gelled multiple emulsions gave better protection to encapsulated hydroxytyrosol during gastrointestinal digestion, compared to conventional emulsions and multiple emulsions [104].

Although substantial advances in the development of multiple emulsions have been made, the wide application of these systems in the food industry is still limited. This is because there are a number of challenges to their commercial application. They are more difficult, costly, and time consuming to prepare than conventional emulsions. They are more susceptible to breakdowns during storage or after exposure to the conditions experienced by many foods, such as shear forces, thermal processing, dehydration, and freezing/thawing. As mentioned earlier, the stability of multiple emulsions can be enhanced by the regulation of osmotic pressure between the aqueous phases, gelation of the aqueous phase, crystallization of the oil phase, and/or coating with colloidal particles. Another challenge is that encapsulated bioactive compounds may be lost from the multiple emulsions during their preparation or storage. For example, the loss of antioxidant activity was reported to be more pronounced in multiple emulsions containing omega-3 oils during preparation and storage than in conventional emulsions [214]. Therefore, the formulation and preparation conditions of multiple emulsions must be carefully selected, depending on the requirements of the particular food products.

## 8. Emulgels and Other Systems

It should be noted that there are a number of other emulsion-based systems that can also be considered to be advanced emulsions [1]. For instance, emulgels are gelled emulsions, which may be formed by incorporating a gelling agent into the continuous phase of an emulsion or by promoting the formation of a network of aggregated droplets. In the latter case, aggregation can be achieved by reducing the repulsion between oil droplets, or by increasing the attraction between them [4]. Emulgels are useful for food applications when a semi-solid or highly viscous texture is required. Another kind of advanced emulsion system that was not covered earlier are filled microgels, which consist of lipid droplets trapped inside small biopolymer spheres [4]. Filled microgels can be used to protect bioactive agents, to control the release of active agents, to control the digestion of macronutrients in the gastrointestinal tract, and as reduced fat systems. Finally, it should be noted that emulsions can also be used as templates to form other kinds of particles. For instance, W/O and O/W/O emulsions can be used as a template to form hydrogel beads or filled hydrogel beads with well-defined sizes, whereas O/W emulsions can be used as a template to form emulsified organogels.

## 9. Conclusions

Although conventional oil-in-water emulsions are currently the most widely used emulsions in the food industry, they are often susceptible to breakdowns over time or when exposed to certain environmental stresses during their production, transport, storage, or utilization. Additionally, they have only a limited capacity to encapsulate, protect, and deliver bioactive components. To this end, there have been major advances in the exploration of advanced emulsion technologies to extend, improve, or create novel functional performances in recent years. This review has focused on the major types of advanced emulsions currently available, including nanoemulsions, high internal phase emulsions, Pickering emulsions, multilayer emulsions, solid lipid nanoparticles, and multiple emulsions. The physicochemical principles and key technical information underlying the preparation of these advanced emulsions, the potential advantages and disadvantages of each of the systems, and the stabilization mechanisms have been discussed through various case studies.

Finally, we should state that advanced emulsions are often expensive and difficult to prepare and are sometimes more unstable than conventional emulsions. There is still a pressing need for commercially viable large-scale preparation procedures and materials for creating advanced emulsions. When applied to deliver bioactive compounds, advanced emulsions feature superior properties over conventional emulsions, associated with higher retention efficiency, increased stability to environmental stresses, improved bioavailability, and the ability to control or trigger the release. However, the full potential in delivering bioactives has still not been reached. Particularly, detailed information on the biological fate of advanced emulsions is still rather limited and is an area where further research is required. Overall, this review article may guide future trends in the development of novel strategies for the fabrication of advanced emulsions that are suitable for food applications.

## Figures and Tables

**Table 1:** Recent examples of the utilization of nanoemulsions as delivery systems for various bioactive compounds.

Bioactive Compounds	Method	Particle Diameter	Results	Ref.
Citral	Sonication	˂100 nm	The citral nanoemulsions showed antimicrobial activity against bacteria	[32]
Anise oil	High pressure homogenization	110–180 nm	Nanoemulsions of anise oil showed better long-term stability and antimicrobial activity than bulk anise oil	[33]
β-carotene	Microfluidization	140–160 nm	At 4 °C and 25 °C, the nanoemulsions remained stable throughout 14 days of storage and retarded the degradation of β-carotene	[34]
β-carotene	Spontaneous emulsification	109–145 nm	The transformation and bioaccessibility of β-carotene in the gastrointestinal tract depended on the lipid phase composition of nanoemulsions	[19]
β-carotene	High pressure homogenization	170–180 nm	Nanoemulsions enhanced β-carotene bioaccessibility and bioavailability	[20]
Lycopene	High pressure homogenization	100–200 nm	Lycopene nanoemulsions were partially (66%) digested and highly bioaccessible (>70%)	[21]
Resveratrol	Spontaneous emulsification	45–220 nm	Encapsulation of resveratrol in nanoemulsions improved its chemical stability after exposure to UV light	[14]
Resveratrol	Sonication	20 nm	Nanoemulsions had good loading, and prevented degradation of resveratrol	[35]
Resveratrol	High pressure homogenization	150 nm	The in vitro release of resveratrol exhibited a sustained release profile and the digestion rate of linseed oil was improved	[22]
Vitamin D_3_	High pressure homogenization	˂200 nm	Whole-fat milk was fortified with vitamin-enriched nanoemulsions and remained stable to particle growth and gravitational separation for ten days	[13]
Vitamin D_3_	High pressure homogenization	˂200 nm	An animal study showed that the coarse emulsions increased the serum 25(OH)D_3_ by 36%, whereas the nanoemulsions significantly increased the serum 25(OH)D_3_ by 73%	[29]
Astaxanthin	Spontaneous emulsification	150–160 nm	Nanoemulsions protected astaxanthin from photodegradation	[36]
Curcumin	High pressure homogenization	80 nm	Nanoemulsions increased the bioaccessibility of curcumin	[23]
Curcumin	Spontaneous emulsification	40–130 nm	Coating with curcumin nanoemulsions can enhance quality and shelf life of chicken fillets	[15]
Curcumin	High pressure homogenization	90–122 nm	Curcumin nanoemulsion-fortified milk exhibited significantly lower lipid oxidation than control (unfortified) milk and milk containing curcumin-free nanoemulsions	[13]
Curcumin	Microfluidization	˂180 nm	Curcumin bioaccessibility was appreciably higher in the presence of nanoemulsions than in their absence	[24]
Curcumin	Microfluidization	83 nm	The droplet size plays a critical role in the degradation of curcumin	[25]
Ginger essential oil	Sonication	57 nm	Ginger essential oil nanoemulsions are used as edible coatings to preserve the quality attributes of chicken breast	[16]
Propolis	Phase inversion emulsification	50 nm	Propolis nanoemulsion can keep the biological activities of extract and be used as a natural food preservative	[37]
5-demethylnobiletin	High pressure homogenization	170–180 nm	The absorption and metabolism of 5-demethylnobiletin depended on oil type in nanoemulsions	[27]
Capsaicin	Sonication	168 nm	Capsaicin nanoemulsion reduced rat gastric mucosa irritation	[13]
Coenzyme Q_10_	Microfluidization	200 nm	The bioavailability of coenzyme Q_10_ nanoemulsion in vivo increased 1.8-fold compared with coenzyme Q_10_ dissolved in oil	[31]

**Table 2:** Recent examples of multilayer emulsions for the delivery of various bioactive compounds.

First Layer	Second Layer	Bioactive Compounds	Results	Refs
Chitosan	Alginate	Curcumin	The biopolymer coating protected curcumin from degradation and preserved its antioxidant capacity during digestion	[23]
Chitosan	Pectin	Astaxanthin	The pectin coating retarded astaxanthin degradation during storage 3- to 4-fold	[98]
Gelatin	Chitosan	Fish oil	The chitosan coating increased emulsion stability during storage and within the gastric phase	[91]
Lactoferrin	Alginate	β-carotene	The alginate coating increased lipid digestibility and β-carotene bioaccessibility	[59]
Lactoferrin	Alginate	Resveratrol	The alginate coating retained the antioxidant activity of resveratrol during storage	[94]
Lactoferrin	Alginate	Curcumin	The alginate coating modulated the rate of lipid digestion and free fatty acid release in a model gastrointestinal tract	[95]
Lactoferrin	Beet pectin	β-carotene	The secondary emulsions were highly stable from pH 3 to 9 due to the thick biopolymer coating formed around the oil droplets	[100]
Lactoferrin	Lactoferrin-polyphenol conjugate	β-carotene	The lactoferrin-EGCG conjugate improved the chemical stability of β-carotene	[101]
OSA starch	Chitosan	β-carotene	The multilayer coating improved β-carotene stability during storage	[97]
Whey protein isolate (WPI)	Alginate	Curcumin	The second layer significantly enhanced the encapsulation efficiency and antioxidant activity of curcumin during 3 weeks of storage	[92]
WPI	Alginate	Flaxseed oil	Sonication and freeze-drying promoted oxidation of flaxseed oil	[93]
WPI	Persian gum	Astaxanthin	Multilayer emulsions improved the stability of the natural color	[104]
WPI	Xanthan-locust bean gum	Fish oil	Multilayer emulsions had high creaming and oxidative stability at 5 mM salt (pH 7.0)	[105]

**Table 3:** Recent examples of Pickering emulsions for the delivery of various bioactive compounds. Key: WPI = whey protein isolate; TPP = tripolyphosphate; EGCG = epigallocatechin gallate; CMC = carboxymethyl cellulose; β-Lg = β-lactoglobulin.

Particles	Particle Formation Method	Bioactive Compounds	Results	Refs
Starch particles	Octenylsuccinate quinoa starch	Lutein	Encapsulation improved the storage stability of lutein, with the half-life times increasing from 12 to 41 days	[149]
Starch particles	Media-milling	Curcumin	Curcumin bioaccessibility increased from 11% in bulk oil to 28% in Pickering emulsions	[147]
Ovotransferrin particles	Genipin cross-linking	Hesperidin	Hesperidin bioaccessibility increased from 55% in bulk oil to 62% in Pickering emulsions	[150]
Kafirin nanoparticles	Extraction from whole sorghum grain	Curcumin	Pickering emulsions had stronger protective effects on curcumin when subjected to UV radiation as compared to Tween 80 stabilized emulsions	[145]
WPI nanogels	Heat denaturation	Curcumin	The partitioning of curcumin in the dispersed phase varied as a function of pH in an in vitro release model with lower partitioning at pH 3.0 as compared to that at pH 7.0	[151]
WPI-lactose-EGCG complexes	Maillard reaction and complexation	Curcumin	Glycated WPI-lactose/EGCG-stabilized emulsions exhibited stronger thermal stability and higher curcumin retention than WPI-stabilized ones	[123]
WPI-chitosan complexes	Polyelectrolyte complexation	Lycopene	Encapsulated lycopene had higher storage stability and sustained release behavior under simulated GIT conditions	[152]
Chitosan-TPP nanoparticles	TPP cross-linking	Curcumin	Curcumin encapsulated in Pickering emulsions exhibited a sustained release profile	[138]
Chitosan-gum arabic nanoparticles	Polyelectrolyte complexation	Curcumin	Pickering emulsions protected curcumin from degradation during storage and controlled its release during in vitro digestion	[153]
CMC-quaternized chitosan complexes	Polyelectrolyte complexation	Curcumin	Pickering emulsions had gel-like behavior, exhibited high stability against environmental stresses, and reduced curcumin degradation	[154]
Zein-chitosan complexes	Antisolvent approach	Curcumin	Pickering emulsions protected curcumin from degradation	[146]
Zein-pectin nanoparticles	Polyelectrolyte complexation	Cinnamon oil	Pickering emulsions exhibited better antibacterial activity than pure essential oils due to their better dispersibility and sustained-release profile	[142]
Ovotransferrin-lysozyme complexes	Polyelectrolyte complexation	Curcumin	Curcumin bioaccessibility was increased from 16% to 38% after encapsulation in Pickering emulsions	[148]
β-Lg-EGCG complexes	Hydrogen bonding/hydrophobic interactions	Lutein	Pickering emulsions protected lutein from degradation during storage	[120]
β-Lg-gum arabic complexes	Polyelectrolyte complexation	Lutein	Pickering emulsions protected lutein from degradation during storage	[155]

**Table 4:** Recent examples of solid lipid nanoparticles (SLNs) for the delivery of various bioactive compounds.

Solid Lipid	Emulsifier	Bioactive Compounds	Results	Refs
Vegetable fat	Soy lecithin	Vitamin D_3_	SLNs retained 86% of vitamin after 65 days, compared to 61% for free vitamin	[163]
Palmitic acid	Egg lecithin/ Span 85	Anthocyanin	SLNs increased the stability of anthocyanins against high pH and temperatures	[170]
Palmitic acid	Whey protein isolate	Fish oil	SLNs effectively inhibited the oxidation of fish oil	[181]
Palmitic acid/ corn oil	Whey protein isolate	β-carotene	WPI increased the colloidal stability of SLNs, and improved β-carotene oxidative stability	[180]
Tristearin	PEGylated	Curcumin	Curcumin in PEGylated SLNs rapidly permeated through the epithelium, conferring a >12-fold increase in bioavailability compared to pure curcumin	[178]
Compritol 888 ATO	Pluronic F68	Curcumin	Parallel artificial membrane permeability assay showed a great increase in curcumin permeation when formulated as SLNs.	[168]
Stearic acid	Sodium caseinate/ pectin	Curcumin	Natural biopolymer-emulsified SLNs were prepared as curcumin delivery systems	[175]
Glyceryl monostearate	Tween 80/span 80	Citral	67% citral retained in SLNs after 12 days, whereas only 8% retained in control	[172]
Cocoa butter	Monoglyceride/ diglyceride/sodium stearoyl-2-lactylate	EGCG	SLNs protected EGCG during storage and under environmental stress	[167]
Glyceryl tristearate	Lecithin	Vitamin E	Vitamin E in SLNs remained stable during storage and its antioxidant activity was maintained	[186]
Witepsol H15	Tween 80	Rosmarinic acid	The bioactivity of rosmarinic acid in SLNs was retained when stored in a N_2_ controlled atmosphere for 365 days	[187]
Glycerol distearate/ glycerol monostearate	Lecithin/tween 80	Lycopene	Lycopene-loaded SLNs used to fortify an orange drink	[171]
Steric acid/tripalmitin	Tween 80/span 80	Curcumin	SLNs prolonged the release of curcumin during 48 h at pH 6.8	[33]
Glycerol monostearate/ steric acid	Soy lecithin	EGCG	EGCG-loaded SLNs exhibited higher anticancer activities than pure EGCG	[167]
Glycerol monostearate/Propylene glycol monopalmitate	Sodium caseinate-lactose Maillard conjugate	Curcumin	SLNs greatly enhanced the antioxidant activity and retention of curcumin during storage	[179]

**Table 5:** Recent examples of multiple emulsions for the delivery of various bioactive compounds. Key: WPC = whey protein concentrate; OSA = octenyl succinate.

Bioactive Compounds	Emulsifier (W_1_/O)	Emulsifier (O/W_2_)	Results	Refs.
Anthocyanin	PGPR	Quillaja saponin	Anthocyanin transfer between aqueous phases depended on pH, temperature, and initial location	[197]
Anthocyanin	PGPR	Gum arabic	Multiple emulsions controlled the release of anthocyanins in the stomach	[199]
Anthocyanin	PGPR	Kafirin nanoparticles	The osmotic pressure-driven swelling process was the major challenge for the long-term stability of Pickering multiple emulsions during storage	[203]
Anthocyanin	PGPR	Tween 20/guar gum	Multiple emulsions exhibited high (91%) encapsulation efficiency and high kinetic stability	[211]
Anthocyanin	Span 80/ tween 20	WPC-gum arabic complexes	WPC-gum arabic complexes improved stability at pH 4.5	[193]
Vitamin B_12_	PGPR	Sodium caseinate	Non-adsorbed PGPR in the oil phase played a key role in emulsion stability	[192]
Vitamin D_3_	Span 80/ lecithin	Chitosan-gum arabic complexes	Calcium ions in the intestinal fluids decreased free fatty acid release and vitamin D_3_ bioaccessibility	[194]
Folic acid	PGPR	WPC-pectin complexes	Optimum conditions were determined as 1% pectin, 4% WPC, and 15% dispersed phase (pH 6.0), with 99% encapsulation efficiency of folic acid	[204]
Gallic acid	PGPR/ Span 80	Tween 80	Multiple emulsions were stable for 28 days and maintained more than 50% of gallic acid antioxidant capacity	[59]
Gallic/ quercetin	PGPR	Sodium caseinate	Multiple emulsions were developed as potential fat replacers	[212]
Fish protein hydrolysate	PGPR	WPC-inulin complexes	Homogenization conditions were optimized to improve stability and encapsulation efficiency	[205]
Soy peptides	PGPR	OSA starch	Freeze-dried emulsion powders had higher encapsulation (>70%) than spray-dried ones	[198]
Casein peptides	PGPR	Sodium caseinate	The release of peptides can be controlled by adjusting oil phase composition	[201]
Resveratrol	PGPR	Tween 20	Optimized emulsions had high encapsulation efficiency (up to 58%) and good storage stability	[83]
Trans-resveratrol	PGPR	Tween 20	Optimized emulsions had high colloidal stability and large trans-resveratrol carrier capacity	[196]
β-sitosterol	PGPR	Tween 20	Emulsions prepared at 300 bar for 3 cycles had the most desirable stability of β-sitosterol	[192]
Oleuropein	Span 80	WPC-pectin complexes	At optimum conditions, a droplet size of 191 nm, zeta potential of −26.8 mV, and encapsulation efficiency of 91% were achieved	[195]

## Data Availability

Data sharing not applicable.
